# Selection and Validation of Induction Chemotherapy Beneficiaries Among Patients With T3N0, T3N1, T4N0 Nasopharyngeal Carcinoma Using Epstein-Barr Virus DNA: A Joint Analysis of Real-World and Clinical Trial Data

**DOI:** 10.3389/fonc.2019.01343

**Published:** 2019-11-29

**Authors:** Cheng Xu, Shu Zhang, Wen-Fei Li, Lei Chen, Yan-Ping Mao, Ying Guo, Qing Liu, Jun Ma, Ling-Long Tang

**Affiliations:** ^1^Department of Radiation Oncology, Sun Yat-sen University Cancer Center, State Key Laboratory of Oncology in South China, Collaborative Innovation Center for Cancer Medicine, Guangdong Key Laboratory of Nasopharyngeal Carcinoma Diagnosis and Therapy, Guangzhou, China; ^2^Clinical Trials Centre, Sun Yat-sen University Cancer Center, State Key Laboratory of Oncology in South China, Collaborative Innovation Center for Cancer Medicine, Guangdong Key Laboratory of Nasopharyngeal Carcinoma Diagnosis and Therapy, Guangzhou, China; ^3^Department of Medical Statistics and Epidemiology, School of Public Health, Sun Yat-sen University, Guangzhou, China

**Keywords:** nasopharyngeal carcinoma, Epstein-Barr virus, induction chemotherapy, concurrent chemoradiotherapy, recursive partitioning analysis

## Abstract

**Background and Purpose:** Evidence for induction chemotherapy plus concurrent chemoradiotherapy (IC+CCRT) in nasopharyngeal carcinoma (NPC) was derived from landmark clinical trials excluding the T3N0, T3N1, T4N0 subgroups. This study used Epstein-Barr virus (EBV) DNA to select IC beneficiaries from the three subgroups.

**Materials and Methods:** Significant predictors of overall survival (OS) were identified using multivariate Cox analyses. Risk stratification was generated using recursive partitioning analysis (RPA). IC+CCRT was compared with CCRT in each risk stratification and in different subgroups. Individual-level data from a clinical trial (NCT01245959) was used for validation.

**Results:** Gender and EBV DNA were included in RPA-generated risk stratification, categorizing patients into low-risk (EBV DNA <2,000 copies/mL; female and EBV DNA ≥2,000 copies/mL) and high-risk groups (male and EBV DNA ≥2,000 copies/mL). The OS superiority of IC+CCRT over CCRT was only observed in the high-risk group (HR = 0.64, 95% CI = 0.43–0.97; *P* = 0.032). Subgroup analysis indicated the OS benefit was exclusively from the docetaxel–cisplatin−5-fluorouracil regimen (HR = 0.41, 95% CI = 0.22–0.78; *P* = 0.005). The status of the T3N1 subgroup as an IC beneficiary is more explicit than the T3N0 and T4N0 subgroups. IC+CCRT showed improved OS in the validation cohort combining high-risk cases of real-world data with clinical trial data (HR = 0.62, 95% CI = 0.42–0.94; *P* = 0.023).

**Conclusion:** Patients with high-risk T3N1 NPC is the definite target population for receiving IC+CCRT in real-world practice. T3N0 and T4N0 subgroups need further investigations in future IC-related studies.

## Introduction

As nasopharyngeal carcinoma (NPC) has the highest incidence in endemic areas such as Southern China, randomized controlled trials (RCTs) conducted in this region are incredibly important in optimizing clinical decision-making (1, 2). In excess of 70% of new cases are defined as locoregionally advanced NPC (LANPC; stage III–IVA), which is prone to distant metastasis and therefore requires intensive treatments over and above radiotherapy alone (3).

Since the INT 0099 trial successfully introduced chemotherapy for improved management of LANPC in 1998, various chemoradiotherapy schedules have been investigated using clinical trials (4–8). In the past two decades, concurrent chemoradiotherapy (CCRT) followed by adjuvant chemotherapy (AC) has been recommended by the National Comprehensive Cancer Network (NCCN) clinical guidelines as the standard treatment for LANPC due to its strong therapeutic intensity (9). However, a clinical trial from endemic area by Chen et al. reported that the additional AC induced severe gastrointestinal toxicities and low patient compliance (63%), which greatly restricted its broad practical application (5). Induction chemotherapy (IC) is used before radiotherapy and thought to be less toxic, improve tumor shrinkage, and lead to early eradication of micrometastases (10). The 2018 NCCN guidelines increased the recommendation of IC+CCRT from category III to IIA as one of the most appropriate treatments for LANPC, rendering it superior to CCRT (IIB) and equivalent to CCRT+AC (IIA) (11).

Three phase III RCTs from endemic areas provided supporting evidences for IC+CCRT (7, 8, 12). Cao et al. (7) investigated the cisplatin−5-fluorouracil (PF) IC regimen in LANPC excluding T3N0–1 subgroup and found IC+CCRT achieved higher 3-year disease-free survival than CCRT alone (82.0 vs. 74.1%; *P* = 0.028). Sun et al. (8) and Zhang et al. (12) individually explored docetaxel–cisplatin−5-fluorouracil (TPF) and gemcitabine–cisplatin (GP) IC regimens in LANPC excluding T3–4N0 subgroups. Both trials suggested that the additional IC can significantly improve 3-year overall survival (OS) compared with CCRT alone. Notably, target population of the three RCTs covered all LANPC but not T3N0–1 (7) or T3–4N0 (8, 12) subgroups, since these patients were crudely considered to have low risk of distant metastasis and not warranting additional IC. Although this inclusion criterion enhanced the power to detect survival benefits of IC+CCRT, it raised clinical questions that whether patients with T3N0, T3N1, and T4N0 NPC could benefit from IC, in that data on a relatively favorable subgroup is scarce and these patients are not always included in clinical trials. A phase III trial including all subgroups of LANPC patients in a non-endemic area reported non-significantly different OS between IC+CCRT and CCRT (*P* = 0.059) (13). Thus, the three subgroups (i.e., T3N0, T3N1, and T4N0) has become a potentially confounding factor that may exert effects on trial results, yet it has not been thoroughly investigated.

As the Tumor-Node-Metastasis (TNM) staging system only utilizes anatomical information, it solely may fail to identify IC beneficiaries from the three excluded subgroups. Epstein-Barr virus (EBV) DNA has been demonstrated to better refine risk stratification and guide individualized treatment in NPC (14). In this retrospective, joint analysis based on real-world and clinical trial data, we used pre-treatment EBV DNA and other critical predictors to select and validate the IC beneficiaries from these excluded T3N0, T3N1, and T4N0 NPC cases, with the purpose of providing real-world evidences to inform choices between treatment strategies in patients with T3N0, T3N1, and T4N0 NPC.

## Materials and Methods

### Study Design, Data Source, and Population

A flow diagram depicting the study design and inclusion/exclusion criteria is presented as Supplementary Figure 1. Given the reliance on a big-data intelligence platform (YiduCloud Technology Ltd., Beijing, China), we generated a NPC-specific real-world dataset that was adopted to identify all untreated, non-metastatic cases that were initially diagnosed at Sun Yat-sen University Cancer Center (SYSUCC) between April 2009 to December 2015. All patients received radical treatments based on intensity-modulated radiotherapy (IMRT) and complete basic data were obtained for each patient. A detailed description of the intelligence platform is presented in Supplementary Materials and has been published in a previous study (15).

This study was approved by the Institutional Review Board and the Ethics Committee with the approval ID YB2018-71; the need for informed consent was waived. To ensure study integrity, original raw data have been uploaded to a public platform named Research Data Deposit (http://www.researchdata.org.cn) with the identifier RDDA2018000782.

### Pre-treatment Workup

Clinical staging was guided by the 8th edition of the American Joint Committee on Cancer/Union for International Cancer Control (AJCC/UICC) manual. Pre-treatment examinations included complete medical history, physical examination, blood profile, nasopharyngoscopy, head-to-neck magnetic resonance imaging (MRI), chest radiography/computed tomography (CT), abdominal ultrasound, and skeletal scintigraphy; ^18^F-fluorodeoxyglucose positron emission tomography-CT was used to replace the latter three items for detection of possible metastases in the lung, liver, and bones. Moreover, circulating cell-free EBV DNA was quantified using a real-time quantitative polymerase chain-reaction (PCR) assay; the detailed method for this has been described in a previous study (14).

### Treatment

All patients underwent IMRT using the simultaneous integrated boost technique on 5 consecutive days every week. IC regimens consisted of PF regimen (80 mg/m^2^ and 4,000 mg/m^2^, respectively), docetaxel–cisplatin (TP; 75 and 75 mg/m^2^, respectively), and TPF (60, 60, and 3,000 mg/m^2^, respectively), every 3 weeks for 2–3 cycles. Concurrent chemotherapy was weekly (30–40 mg/m^2^) or 3-weekly cisplatin (80–100 mg/m^2^) treatment. Detailed information is shown in the Supplementary Materials.

### Follow-Up and Endpoints

Follow-up duration was measured from the day of diagnosis to the last visit or death. During the visits, head-to-neck MRI, chest radiography/CT, abdominal ultrasound, and skeletal scintigraphy were routinely performed, every 3 months during the first 2 years, then every 6 months for 3 years thereafter. Clinical suspicion of recurrence and distant metastasis were confirmed using cytological biopsies and imaging. The main endpoint was OS, measured from day of diagnosis until death due to any cause or the latest known date alive. Secondary endpoints were failure-free survival (FFS; from the date of diagnosis to failure, death, or last follow-up), locoregional relapse-free survival (LRRFS; to local/regional relapse), and distant metastasis-free survival (DMFS; to distant metastasis).

### Statistical Analysis

Continuous variables were converted into categorical variables based on the interquartile range (IQR; age at diagnosis) and clinical cut-off values [hemoglobin (Hb), albumin, lactate dehydrogenase (LDH), and C-reactive protein (CRP)]. Robust evidence has indicated that pre-treatment EBV DNA can refine the TNM staging for NPC at the cut-off of 2,000 copies/mL (16), which was also supported by this study using the receiver-operating characteristic (ROC) curve analysis (Supplementary Figure 2). Actuarial survival rates were calculated using the Kaplan-Meier curve and compared using the log-rank test (17). Univariate and multivariate Cox regression models were performed to quantify the effect of variables on OS. Univariate Cox analysis was performed *a priori* via a hypothesis-driven method. Predictors with *P* < 0.05 in the univariate analysis were entered into the multivariate analysis to validate their significance by backward stepwise algorithm (18). Hazard ratios (HRs) and 95% confidence intervals (CIs) were used as the summary statistics.

In accordance with the optimized binary partition algorithm, we included all validated predictors for 5-year OS to perform recursive partitioning analysis (RPA) using the *rpart* package in R, with the purpose of distinctly categorizing heterogeneous patients into purified risk stratifications (19). The *prune* package in R was used to remove the excessive branches of RPA-generated risk stratification for realistic application (19).

Individual-level, 5-year follow-up data of the TPF trial (NCT01245959), which discarded the T3–4N0 NPC cases was used to establish validation cohorts (20). Essentially, we aim to use the real-world dataset of T3–4N0 NPC patients to establish pseudo-trial cohorts (basic characteristics of patients should be consistent with their counterparts in a clinical trial), combine these data with the trial data, and determine whether the original trial results have changed significantly. Consequently, we validate the importance of additional IC to patients with T3–4N0 NPC. A three-step method was used to achieve this. Firstly, patients with T3–4N0 NPC at different risks were individually selected from the real-world dataset to produce two pseudo-trial cohorts. The sample size of T3–4N0 NPC was estimated according to its proportion, relative to the whole NPC population. Secondly, pseudo-trial cohorts of T3–4N0 NPC were processed to have similar baseline characteristics to the TPF trial, by using propensity score matching (PSM) to balance potential differences, since PSM can excellently mimic features of clinical trials and reduce selection bias caused by observed confounders (21). PSM was used in accordance with the nearest-neighbor algorithm without replacement. Thirdly, two validation cohorts were generated by combining pseudo-trial cohorts with the TPF trial data and verified by Kaplan-Meier survival analysis. C-index was used to measure the discriminatory performance of treatment via the *Hemins* package in R. All statistical analyses and figures were generated using SPSS, version 23.0 (SPSS Inc., Chicago, IL, USA) and R software, version 3.3.2 (http://www.r-project.org/). All tests were two-sided; *P* < 0.05 was significant.

## Results

### Baseline Characteristics and Survival of Patients With T3N0, T3N1, T4N0 NPC

The baseline characteristics of 2,692 patients with T3N0, T3N1, and T4N0 NPC are shown in Table 1. The median age was 45 (IQR = 37–52) years, with a male-to-female ratio of 2.6:1.0. Non-keratinizing undifferentiated NPC (World Health Organization type III) accounted for the majority (97.5%) of all endemic cases. The proportion of patients with pre-treatment EBV DNA ≥2,000 copies/mL was 57.5%.

**Table 1:** Baseline characteristics of patients with T3N0, T3N1, and T4N0 NPC.

**Characteristics**	**No. (%)**	**Univariate analysis of OS**		**Multivariate analysis of OS**	
		**HR (95% CI)**	***P***	**aHR (95% CI)**	***P***
Age at diagnosis, years					
18–37	528 (19.6)	Reference		Reference	
38–44	769 (28.6)	1.48 (0.92–2.38)	0.108	1.42 (0.88–2.29)	0.148
45–52	655 (24.3)	1.10 (0.66–1.85)	0.715	1.12 (0.66–1.88)	0.678
≥53	740 (27.5)	2.38 (1.52–3.74)	<0.001	2.10 (1.32–3.31)	0.001
Gender					
Male	1,944 (72.2)	Reference		Reference	
Female	748 (27.8)	0.58 (0.40–0.84)	0.004	0.60 (0.41–0.87)	0.007
Histological type					
WHO type I–II	65 (2.4)	Reference		Reference	
WHO type III	2,626 (97.5)	0.34 (0.20–0.59)	<0.001	0.35 (0.20–0.60)	<0.001
Family history of cancer					
No	1,944 (72.2)	Reference		–	–
Yes	748 (27.8)	0.83 (0.60–1.16)	0.276	–	–
Comorbidity					
No	1,510 (56.1)	Reference		–	–
Yes	1,182 (43.9)	1.33 (0.98–1.79)	0.067	–	–
Cigarette smoking					
No	1,757 (65.3)	Reference		–	–
Yes	935 (34.7)	1.22 (0.91–1.63)	0.186	–	–
Alcohol consumption					
No	2,325 (86.4)	Reference		–	–
Yes	367 (13.6)	1.28 (0.87–1.89)	0.215	–	–
EBV DNA titer, copy/mL^a^					
<2,000	1,547 (57.5)	Reference		Reference	
≥2,000	1,145 (42.5)	2.09 (1.56–2.81)	<0.001	1.96 (1.45–2.64)	<0.001
Hb, g/L^a^^,^^b^					
<120.0 (110.0)	75 (2.8)	Reference		–	–
≥120.0 (110.0)	2,617 (97.2)	1.75 (0.82–3.73)	0.147	–	–
Albumin, g/L^a^					
<40.0	196 (7.3)	Reference		Reference	
≥40.0	2,496 (92.7)	0.42 (0.28–0.64)	<0.001	0.54 (0.35–0.82)	0.011
LDH, U/L^a^					
≤250	2,507 (93.1)	Reference		Reference	
>250	185 (6.9)	2.25 (1.51–3.37)	<0.001	2.00 (1.33–3.01)	0.001
CRP, mg/L^a^					
≤3.00	1,835 (68.2)	Reference		Reference	
>3.00	857 (31.8)	1.55 (1.16–2.06)	0.003	1.19 (0.88–1.60)	0.260
UICC/AJCC clinical stage					
T3N0	401 (14.9)	–	–	–	–
T3N1	2,098 (77.9)	–	–	–	–
T4N0	193 (7.2)	–	–	–	–
T category					
T3	2,499 (92.8)	Reference		Reference	
T4	193 (7.2)	2.30 (1.53–3.46)	<0.001	2.22 (1.47–3.34)	<0.001
N category					
N0	594 (22.1)	Reference		–	–
N1	2.098 (77.9)	0.84 (0.60–1.18)	0.313	–	–
Treatment					
CCRT	1,418 (52.7)	–	–	–	–
IC+CCRT	1,274 (47.3)	–	–	–	–

*NPC, nasopharyngeal carcinoma; OS, overall survival; no., number; HR, hazard ratio; aHR, adjusted hazard ratio; CI, confidence interval; WHO, World Health Organization; EBV, Epstein-Barr virus; Hb, hemoglobin; LDH, serum lactate dehydrogenase; CRP, C-reactive protein; UICC, Union for International Cancer Control; AJCC, American joint Committee on Cancer; CCRT, concurrent chemoradiotherapy; IC, induction chemotherapy*.

a *All of these variables were measured before treatment*.

b *Cut-off values of hemoglobin are 120 and 110 g/L for male and female, respectively*.

In the whole real-world dataset (*N* = 9,354), all survival curves were significantly disparate except for the comparison of stage II and the overall subgroups of T3N0, T3N1, and T4N0 NPC, which had equivalent OS, FFS, LRRFS, and DMFS (all *P* ≥ 0.063; Supplementary Figure 3), indicating a good prognosis for T3N0, T3N1, and T4N0 NPC as a whole. As shown in Table 2, T3N1 had equivalent OS (*P* = 0.116) and LRRFS (*P* = 0.097) compared with T3N0, and equivalent DMFS (*P* = 0.511) compared with T4N0, suggesting homogeneity among patients with T3N0, T3N1, and T4N0 NPC.

**Table 2:** Detailed treatment and prognosis of 2,692 patients with T3N0, T3N1, and T4N0 NPC.

**Items**	**T3N0, no. (%)**	**T3N1, no. (%)**	**T4N0, no. (%)**
No. of patients	401	2,098	193
Treatment			
CCRT	274 (68.3)	1,078 (51.4)	66 (34.2)
IC + CCRT	127 (31.7)	1,020 (48.6)	127 (65.8)
CCRT schedule			
3-weekly	280 (69.8)	1,548 (73.8)	155 (80.3)
Weekly	121 (30.2)	550 (26.2)	38 (19.7)
Accumulated DDP, mg	187	194	188
IC regimen			
TPF	40 (31.5)	454 (44.5)	68 (53.5)
PF	31 (24.4)	232 (22.7)	26 (20.5)
TP	56 (44.1)	334 (32.7)	33 (26.0)
Death (5th yr.)	16 (4.0)	128 (6.1)	23 (11.9)
Locoregional relapse (5th yr.)	15 (3.7)	123 (5.9)	20 (10.4)
Distant metastasis (5th yr.)	11 (2.7)	152 (7.2)	16 (8.3)
Bone	3	18	3
Lung	6	40	3
Liver	0	35	6
Multiple sites	2	45	3
Others	0	14	1
3-year OS (%)	97.3	95.7	91.2
5-year OS (%)	94.3	91.9	85.2
	Ref.	*P* = 0.116	*P* = 0.001
	–	Ref.	*P* = 0.002
3-year FFS (%)	93.5	88.5	83.3
5-year FFS (%)	90.6	84.9	76.6
	Ref.	*P* = 0.003	*P* < 0.001
	–	Ref.	*P* = 0.014
3-year LRRFS (%)	96.2	94.8	91.5
5-year LRRFS (%)	95.6	93.0	85.7
	Ref.	*P* = 0.097	*P* = 0.002
	–	Ref.	*P* = 0.013
3-year DMFS (%)	97.5	93.5	92.0
5-year DMFS (%)	96.8	91.6	90.6
	Ref.	*P* = 0.002	*P* = 0.003
	–	Ref.	*P* = 0.511

*CCRT, concurrent radiochemotherapy; DDP, cisplatin; DMFS, distant metastasis-free survival; FFS, failure-free survival; IC, induction chemotherapy; LRRFS, locoregional relapse-free survival; NPC, nasopharyngeal carcinoma; No., number; OS, overall survival; PF, cisplatin−5-fluorouracil; Ref., reference; TPF, docetaxel–cisplatin−5-fluorouracil; TP, docetaxel–cisplatin; yr., year*.

### RPA-Generated Risk Stratification

After adjustment in multivariate analysis, age (*P* = 0.001), gender (*P* = 0.007), histological type (*P* ≤ 0.001), EBV DNA (*P* ≤ 0.001), albumin (*P* = 0.011), LDH (*P* = 0.001), and T category (*P* ≤ 0.001) were validated to have significant effects on OS (Table 1 and Supplementary Figure 4). All validated predictors were included in RPA to generate risk stratification. After modification of branches based on automatic *rpart* algorithms, gender and EBV DNA were retained in the final model while inessential factors were discarded.

**Figure 1:**
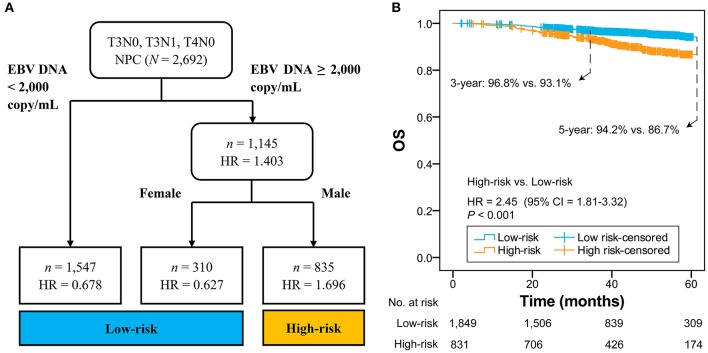
RPA-generated risk stratification **(A)** and the comparison between high-risk and low-risk groups **(B)**. RPA, recursive partitioning analysis; NPC, nasopharyngeal carcinoma; EBV, Epstein-Barr virus; HR, hazard ratio; CI, confidence interval; OS, overall survival.

Figure 1A shows that 2,692 patients with T3N0, T3N1, and T4N0 NPC were categorized into two groups: low-risk group (*n* = 1,857; EBV DNA titer <2,000 copies/mL, female & EBV DNA titer ≥2,000 copies/mL) and high-risk group (*n* = 835; male & EBV DNA titer ≥2,000 copies/mL). The low-risk group had significantly higher OS compared with the high-risk group (HR = 2.45, 95% CI = 1.81–3.32; *P* < 0.001; Figure 1B). Patients with different EBV DNA status in the low-risk group had comparable OS (*P* = 0.739; Supplementary Figure 5).

### Selection of IC Eneficiaries

**Figure 2:**
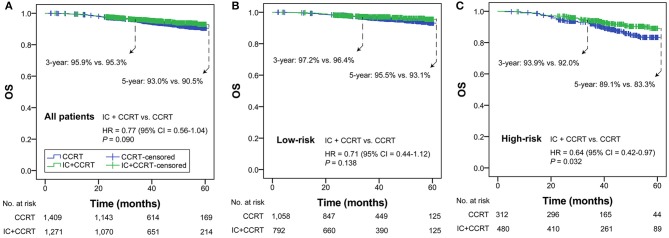
Kaplan-Meier OS curves of IC+CCRT vs. CCRT in the overall T3N0, T3N1, and T4N0 NPC **(A)**, low- **(B)** and high-risk groups **(C)**. HR, hazard ratio; CI, confidence interval; OS, overall survival; IC, induction chemotherapy; CCRT, concurrent chemoradiotherapy.

In all patients with T3N0, T3N1, T4N0 NPC, OS was not significantly different in the comparison of IC+CCRT and CCRT (HR = 0.77, 95% CI = 0.56–1.04; *P* = 0.090). In the low-risk group, a non-significant difference in OS was observed between IC+CCRT and CCRT (HR = 0.71, 95% CI = 0.44–1.12; *P* = 0.138). In the high-risk group, patients receiving IC+CCRT had significantly improved OS compared with their counterparts receiving CCRT alone (HR = 0.64, 95% CI = 0.42–0.97; *P* = 0.032; Figures 2A–C).

### Subgroup Analysis

**Figure 3:**
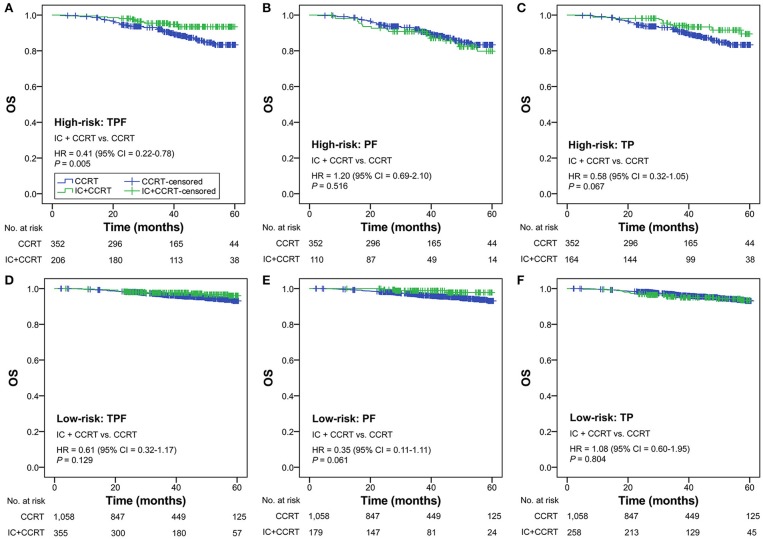
IC+CCRT vs. CCRT in the subgroup analysis based on IC regimen [high-risk: TPF **(A)**, PF **(B)**, TP **(C)**; low-risk: TPF **(D)**, PF **(E)**, TP **(F)**] without adjustment. IC, induction chemotherapy; CCRT, concurrent chemoradiotherapy; TPF, docetaxel–cisplatin−5-fluorouracil; PF, cisplatin−5-fluorouracil; TP, cisplatin−5-fluorouracil.

**Figure 4:**
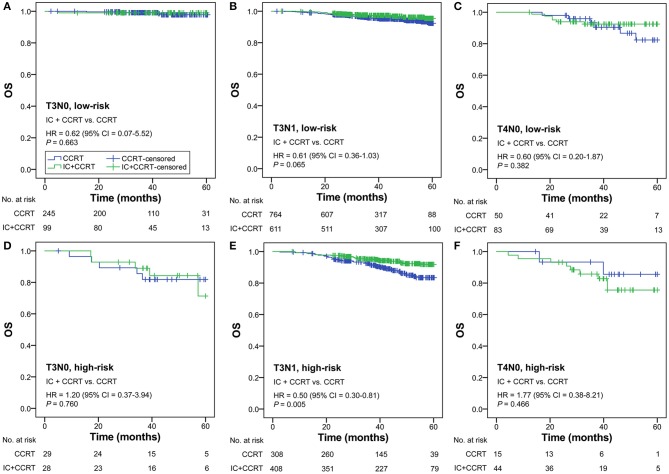
IC+CCRT vs. CCRT in the subgroup analysis based on specific clinical stages [low-risk: T3N0 **(A)**, T3N1 **(B)**, T4N0 **(C)**; high-risk: T3N0 **(D)**, T3N1 **(E)**, T4N0 **(F)**]. IC, induction chemotherapy; CCRT, concurrent chemoradiotherapy.

Subgroup analysis was performed primarily based on IC regimens and specific LANPC subgroups. The improved, non-adjusted OS of IC+CCRT compared with CCRT was only observed in high-risk patients undergoing TPF (HR = 0.41, 95% CI = 0.22–0.78; *P* = 0.005) but not PF, TP, or any of the IC regimens in the low-risk group (all *P* ≥ 0.061; Figure 3). Subgroup analysis was individually performed based on T3N0, T3N1, and T4N0 subgroups. Regardless of the specific clinical stages, low-risk patients treated by IC+CCRT generally had equivalent OS compared with those treated by CCRT alone (all *P* ≥ 0.065). In high-risk patients, IC+CCRT was found to have significant survival benefit in OS compared with CCRT in the T3N1 subgroup (*P* = 0.005), but not in the T3N0 or T4N0 subgroups (Figure 4). The sample size of each treatment arm in T3N0 and T4N0 subgroups was very small, ranging from 15 to 44.

The RPA-generated risk stratification showed superb discriminatory ability in all subgroups, except for T4 category (*P* = 0.065; Table 3). After adjustment of covariates, the superiority of IC+CCRT over CCRT in OS was observed in the high-risk subgroup of age ≥53 years (*P* = 0.006), LDH ≤ 250 (*P* = 0.001), T3 category (*P* = 0.005), N1 category (*P* = 0.003), and TPF IC regimen (*P* = 0.003).

**Table 3:** Subgroup analysis of RPA-generated risk stratification and IC+CCRT vs. CCRT with adjustment.

**Variables**	**High risk vs. low risk**		**IC+CCRT vs. CCRT**			
			**High risk**		**Low risk**	
	**HR (90% CI)**	***P***	**aHR^**a**^ (90% CI)**	***P***	**aHR^**a**^ (90% CI)**	***P***
Age, year						
18–37	3.30 (1.43–7.64)	0.005	0.94 (0.30–3.01)	0.922	0.81 (0.22–3.01)	0.749
38–44	2.81 (1.53–5.18)	0.001	0.47 (0.21–1.04)	0.061	0.83 (0.31–2.18)	0.700
45–52	3.29 (1.61–6.71)	0.001	0.89 (0.34–2.34)	0.810	1.11 (0.36–3.39)	0.862
≥53	1.71 (1.07–2.73)	0.024	0.34 (0.16–0.73)	0.006	0.50 (0.23–1.07)	0.076
Albumin, g/L						
<40.0	2.30 (1.02–5.18)	0.044	0.42 (0.14–1.25)	0.117	0.56 (0.13–2.39)	0.433
≥40.0	2.40 (1.73–3.33)	<0.001	0.67 (0.43–1.06)	0.085	0.78 (0.47–1.28)	0.320
LDH, U/L						
≤250	2.33 (1.68–3.25)	<0.001	0.44 (0.27–0.71)	0.001	0.79 (0.48–1.29)	0.343
>250	2.43 (1.09–5.46)	0.026	2.61 (0.75–9.08)	0.132	0.13 (0.02–1.02)	0.057
T category						
T3	2.51 (1.80–3.49)	<0.001	0.53 (0.34–0.83)	0.005	0.72 (0.43–1.21)	0.212
T4	2.16 (0.95–4.91)	0.065	1.80 (0.38–8.46)	0.456	0.60 (0.18–2.07)	0.422
N category						
N0	5.37 (2.85–10.11)	<0.001	1.42 (0.58–3.48)	0.444	0.74 (0.27–2.06)	0.567
N1	2.02 (1.43–2.85)	<0.001	0.48 (0.30–0.79)	0.003	0.65 (0.38–1.12)	0.119
IC regimen						
TPF	2.34 (1.63–3.35)	<0.001	0.38 (0.20–0.72)	0.003	0.62 (0.32–1.21)	0.159
PF	3.15 (2.19–4.54)	<0.001	1.16 (0.66–2.02)	0.613	0.37 (0.11–1.18)	0.092
TP	2.28 (1.60–3.24)	<0.001	0.58 (0.32–1.06)	0.078	1.10 (0.60–2.02)	0.757

*RPA, recursive partitioning analysis; CCRT, concurrent radiochemotherapy; IC, induction chemotherapy; HR, hazard ratio; aHR, adjusted hazard ratio; LDH, lactate dehydrogenase; TPF, docetaxel–cisplatin−5-fluorouracil; PF, cisplatin−5-fluorouracil; TP, docetaxel–cisplatin*.

a *aHR was adjusted for age, albumin, LDH, T category, N category, and IC regimen, except for the variable that is being analyzed*.

### Validation of the Cohorts Based on Real-World and Clinical Trial Data

**Figure 5:**
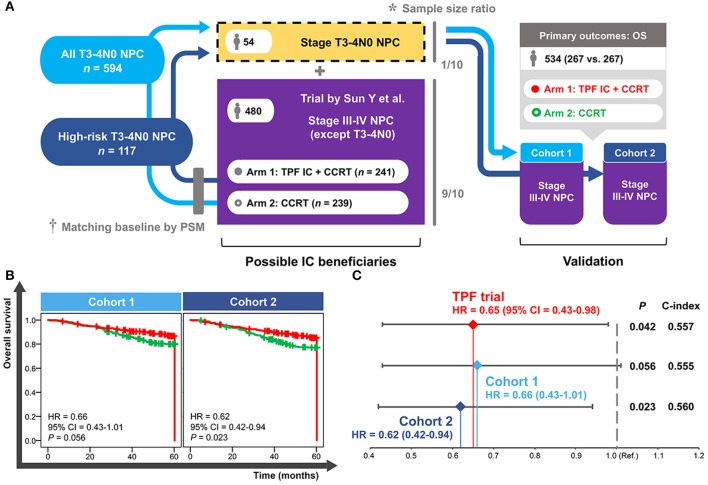
Establishment **(A)** and validation **(B,C)** of the cohorts based on real-world and clinical trial data. *Sample size ratio was calculated based on the NPC-specific real-world dataset including 10,126 patients. ^†^Baseline of the selected T3–4N0 NPC patients was matched with the trial data using the PSM method. NPC, nasopharyngeal carcinoma; PSM, propensity score matching; TPF, docetaxel–cisplatin−5-fluorouracil; IC, induction chemotherapy; CCRT, concurrent chemoradiotherapy; OS, overall survival; HR, hazard ratio; CI, confidence interval; Ref., reference.

A total of 54 patients with T3–4N0 NPC was required to be incorporated into the TPF trial (*n* = 480) in accordance with the sample size ratio of 1 to 9. Eligible patients were individually selected from all of the T3–4N0 NPC group (*n* = 594) and high-risk T3–4N0 NPC group (*n* = 117) to match the trial baselines (e.g., TPF IC regimen, 3-weekly concurrent cisplatin, accumulated concurrent cisplatin ≥200 mg, age, and gender) and produce the validation cohort 1 and cohort 2, respectively. Both cohorts contained 534 patients comparing IC+CCRT with CCRT in LAPNC (267 vs. 267; Figure 5A).

As shown in Figure 5B, a significantly improved OS of IC+CCRT compared with CCRT was only observed in the validation cohort 2 (HR = 0.62, 95% CI = 0.42–0.94; *P* = 0.023) but not cohort 1(HR = 0.66, 95% CI = 0.43–1.01; *P* = 0.056). Cohort 2 showed more obvious superiority of IC+CCRT over CCRT (HR: 0.62 vs. 0.65) and better discrimination performance (c-index: 0.560 vs. 0.557) than the long-term results of the TPF trial (Figure 5C).

## Discussion

With an increasing emphasis on IC in the NCCN guidelines, more robust studies are required to consider evidence on the comparison of IC+CCRT and CCRT in T3N0, T3N1, and T4N0 NPC, given that these patients had been excluded from a majority of IC-related trials and the evidence regarding optimal treatment strategy is limited. In December 2016, the U.S. Congress enacted $$*The 21st Century Cures Act*, which modified the Food and Drug Administration policies and inspired investigators to provide real-world evidence as supplements to clinical trials, in order to expedite the approval process for innovative research (22). This retrospective, joint analysis based on real-world and clinical trial data is the first attempt to identify IC beneficiaries among patients with T3N0, T3N1, and T4N0 NPC based on EBV DNA status (Supplementary Figure 6). We provide robust real-world evidence that can further complement the contemporary trial results.

The 8th edition AJCC/UICC staging system has successfully incorporated human papillomavirus infection status into the TNM classification of oropharyngeal carcinoma (23), which highlights the possibility of including non-anatomic factors to better differentiate the prognosis of EBV-related NPC. EBV is exclusively detected in tumor cells but not in normal nasopharyngeal epithelium; its cell-free DNA has the same polymorphism as the primary lesion tumor and is considered to be released into the peripheral circulation along with the tumor cells death of Lin et al. (24). Previous studies reported that circulating EBV DNA correlate with the tumor burden, stage classification, and survival of patients with NPC (25–27). The practical application of EBV DNA has expanded from initial diagnosis, detection of metastasis, to population screening and pre-treatment risk stratification (28–30). Only two recent studies have included incorporation of pre-treatment plasma EBV DNA into the 8th edition of TNM staging system (16, 31). These studies indicated that the risk of T3N0, T3N1, and T4N0 NPC could be refined by EBV DNA, since both plans covered the three subgroups. A previous retrospective study reported that patients with T3–4N0–1 NPC receiving CCRT could not benefit from additional IC, which may be influenced by the fact that EBV DNA was not used for the screening of IC beneficiaries (32). Similarly, in this study, we reported a non-significant difference in OS between IC+CCRT and CCRT in the whole patients with T3N0, T3N1, and T4N0 NPC when they had not been stratified (Figure 2A). Therefore, high level EBV DNA may be an indicator for physicians to employ IC in LANPC.

Another validated predictor used for risk stratification in this study is gender. Although both genders had the same improved level of plasma EBV DNA, female patients obtained better OS benefits than males (Supplementary Figure 5). This result was in line with a previous finding, which reported that female is associated with better prognosis in NPC compared with male gender (33). One proposed hypothesis is that female hormones can promote immunological responses and confer higher resistance to oxidative damage (34).

This study demonstrated that the OS benefit for high-risk patients was mainly associated with the TPF regimen but not PF or TP IC regimens. The potent triple agent-based TPF regimen has been shown to be a promising prospect in LANPC, allowing patients to receive stronger intensity treatment, longer hospitalization, improved nursing care, and more supportive therapy than the PF regimen, while intensive management itself can lead to better prognosis (8, 13). In addition, the subgroup analysis based on specific clinical stages only supported the high-risk T3N1 subgroup, but not the T3N0 or T4N0 subgroups, as an IC beneficiary. This result should be regarded with caution, since statistical non-significance may be related to the insufficient sample size of two treatment arms in the high-risk T3N0 (28 vs. 29) and T4N0 (44 vs. 15) subgroups. The PF trial that included all LANPC subgroups except T3N0–1 NPC has recently been updated. It revealed a significant 5-year OS benefit of IC+CCRT compared with CCRT (80.8 vs. 76.8%; *P* = 0.04) (35), indicating that the T4N0 subgroup should receive IC+CCRT in clinical practice. Hence, the T3N1 subgroup may not be the only IC beneficiary, and all the three subgroups (T3N0, T3N1, T4N0) should be fully investigated. Since individual patient data of the PF trial is not accessible, we only performed the validation analysis using the 5-year data of the TPF trial (20), which successfully verified the effectiveness of RPA-generated risk stratification.

Several limitations to this study should be stated. First, it is important to recognize that different centers adopt different EBV DNA cut-off values, such as 4,000 copies/mL (36), 500 copies/mL (31), and 1,500 copies/mL (24). Moreover, the heterogeneity in PCR-based EBV DNA testing itself is an important problem, with sensitivity ranging from 53 to 96% (37). Assay harmonization of EBV DNA detection is a major hurdle that has to be overcome prior to incorporation of plasma EBV DNA as a clinical decision-making tool. In 2015, a workshop on harmonization of EBV testing for NPC was hosted by the National Cancer Institute. It offered valuable strategies for establishment of harmonized EBV DNA assays and key recommendations guiding future clinical use (37). Second, in this study, results were driven by the T3N1 subgroup (78%), and the OS superiority of IC+CCRT over CCRT in high-risk patients was only observed in the subgroup of T3 and N1 category. Although validation analysis confirmed that patients with high-risk T3–4N0 NPC could effectively benefit from IC, the sample size of T3N0 and T4N0 subgroups was too small to generate a reliable conclusion. Two robust phase 3 RCTs including all LANPC subgroups except T3–4N0 NPC had reported significant OS improvement of 6.0 and 4.3% from the additional TPF (8) and GP (12) IC regimens, respectively. Therefore, the status of the T3N1 subgroup as an IC beneficiary is more explicit than the T3N0 and T4N0 subgroups, while the latter two require more supporting evidences beyond this study. Third, the retrospective nature limits this study to some extent. This study was performed based on the 8th edition of the AJCC/UICC staging system for a better generalizability in real-world clinical practice. Although the clinical trial in validation analysis used the 7th edition of the AJCC/UICC staging system, the difference in staging systems was too subtle to exert obvious influence on results. Re-staging was not performed in this study because the transform of the staging system from the 7th edition into the 8th edition would compromise data integrity. Nonetheless, this real-world study offers essential information to clinical physicians and trialists, helping them make precise clinical decisions and refine future trial design.

## Conclusion

RPA-generated risk stratification based on pre-treatment plasma EBV DNA provides good and robust efficacy of OS prediction in T3N0, T3N1, and T4N0 NPC. In comparison with CCRT, IC+CCRT leads to significantly improved OS for patients with high-risk T3N1 NPC, which is mainly due to the TPF IC regimen. Patients with high-risk T3N1 NPC is the definite target population for receiving IC+CCRT in real-world practice. T3N0 and T4N0 subgroups need further investigations in future IC-related studies.

## Data Availability Statement

The datasets for this study can be found in the public platform named Research Data Deposit (http://www.researchdata.org.cn) with the identifier RDDA2018000782.

## Ethics Statement

The studies involving human participants were reviewed and approved by Institutional Review Board and the Ethics Committee of Sun Yat-sen University Cancer Center. Written informed consent for participation was not required for this study in accordance with the national legislation and the institutional requirements.

## Author Contributions

L-LT and CX: conceptualization. CX, QL, and YG: methodology. CX, LC, and Y-PM: software. CX and W-FL: validation. CX, SZ, and LC: formal analysis. SZ, YG, and Y-PM: investigation. W-FL and JM: resources. SZ and W-FL: data curation. CX and SZ: writing—original draft preparation. W-FL, JM, and L-LT: writing—review and editing. CX: visualization. L-LT: supervision, project administration, and funding acquisition.

### Conflict of Interest

The authors declare that the research was conducted in the absence of any commercial or financial relationships that could be construed as a potential conflict of interest.
