# Supplementary material for: Selection and Validation of Induction Chemotherapy Beneficiaries Among Patients With T3N0, T3N1, T4N0 Nasopharyngeal Carcinoma Using Epstein-Barr Virus DNA: A Joint Analysis of Real-World and Clinical Trial Data
Source: Front Oncol. 2019 Nov 29;9:1343. doi: 10.3389/fonc.2019.01343 (PMC6896227; doi:10.3389/fonc.2019.01343)
Supplement: Supplementary file 1 [file Data_Sheet_1.docx]

**Methods**

***Study design, data source and population***

A flow diagram depicting the study design and inclusion/exclusion criteria is presented as Supplementary Figure S1. Given the reliance on a big-data intelligence platform (YiduCloud Technology Ltd., Beijing, China), we generated a NPC-specific real-world dataset. This platform was established by Sun Yat-sen University Cancer Center (SYSUCC) in 2015, and has generated 13 disease-specific databases, involving NPC, lung cancer, gastric cancer, colon cancer, and other cancers. The NPC-specific dataset was adopted to identify all untreated, non-metastatic cases that were initially diagnosed at SYSUCC between April 2009 to December 2015. All patients received radical treatments based on intensity-modulated radiotherapy (IMRT) and complete basic data were obtained for each patient.

Based on a well-designed data model and algorithm, the novel big-data research system enables the organization, integration, re-structuring, and updating of real-time data automatically from 20 primary and 8 secondary source systems, resulting in more than 200 million data messages. Patients’ electronic information on demographics (electronic medical records), diagnostics (laboratory test, imaging examination, endoscopy, ultrasound, pathology) and therapeutics (radiotherapy, chemotherapy, supporting therapy) were obtained automatically by the intelligence platform using search terms, such as “diagnosis”, “histology type”, “age at diagnosis”, “gender”, “disease stage”, “radiotherapy technology”, “regimens of chemotherapy”, and “EBV DNA”. Except for the platform’s own stringent online check for data correctness, it allows manual double checking to avoid miscoding and generation of erroneous data, so that all aforementioned data can be easily and safely exported into Microsoft Excel for processing. The database is continuously growing, being updated and maintained by an assigned team, including a physician, programmer, and engineer. As SYSUCC is located in Southern China, this real-world dataset represents a specific subset of endemic cases. A detailed description of the intelligence platform has been published in a previous study (***Lv JW, Chen YP, Huang XD, Zhou GQ, Chen L, Li WF, et al. Hepatitis B virus screening and reactivation and management of patients with nasopharyngeal carcinoma: A large-scale, big-data intelligence platform-based analysis from an endemic area. Cancer 2017; 123: 3540-9.***).

This study was approved by the Institutional Review Board and the Ethics Committee with the approval ID YB2018-71; the need for informed consent was waived. To ensure study integrity, original raw data have been uploaded to a public platform named Research Data Deposit (http://www.researchdata.org.cn) with the identifier RDDA2018000782.

***Treatment***

A stratified multi-therapeutic protocol based on the 8th edition of the AJCC/UICC staging system was employed for patients with nasopharyngeal carcinoma (NPC). All patients were treated with radical radiotherapy using intensity-modulated radiotherapy (IMRT) for the entire course. Patients with stage I NPC received IMRT alone and patients with stage II NPC received IMRT, with or without concurrent chemotherapy. CCRT or induction chemotherapy (IC) plus CCRT were administered to patients with locoregionally advanced NPC (stage III–IVA). Patients receiving CCRT plus adjuvant chemotherapy were excluded from this study, since this approach has been proven to be inferior to CCRT alone, both resulting in severe toxicities and low compliance (***Chen L, Hu CS, Chen XZ, Hu GQ, Cheng ZB, Sun Y, et al. Concurrent chemoradiotherapy plus adjuvant chemotherapy versus concurrent chemoradiotherapy alone in patients with locoregionally advanced nasopharyngeal carcinoma: a phase 3 multicentre randomised controlled trial. Lancet Oncol 2012;13:163-71.***)

Target volumes were delineated slice-by-slice on treatment planning CT scans using an individualized delineation protocol in accordance with the International Commission on Radiation Units and Measurements reports 50 and 62. The prescribed doses were 66–72 Gy in 28–33 fractions to the planning target volume (PTV) of the primary gross tumor volume (GTVnx), 64–70 Gy/28–33 fractions to the PTV of the GTV of the involved lymph nodes (GTVnd), 60–63 Gy/28–33 fractions to the PTV of the high-risk clinical target volume (CTV1), and 54–56 Gy/28–33 fractions to the PTV of the low-risk clinical target volume (CTV2). All targets were treated using the simultaneous integrated boost technique. CTV1 extended 5–10 mm beyond the margin of the GTVnx for potential microscopic spread, including the entire nasopharyngeal mucosa and 5 mm into the submucosal region. CTV2 extended 5–10 mm beyond the margin of the CTV1, potentially involving regions and lymphatic regions, unless the CTV2 was adjacent to critical organs, e.g., brain stem and spinal cord, in which case the extension distance was reduced to 3–5 mm.

IC regimens consisted of platinum-based double or triple agents, namely cisplatin–5-fluorouracil (80 mg/m^2^ and 4,000 mg/m^2^, respectively), docetaxel–cisplatin (75 mg/m^2^ and 75 mg/m^2^, respectively), and docetaxel–cisplatin–5-fluorouracil (60 mg/m^2^, 60 mg/m^2^, and 3,000 mg/m^2^, respectively), every three weeks for 2–3 cycles. All chemotherapeutic drugs were administered on day 1 of each 21-day cycle, except for 5-fluorouracil which was given via continuous intravenous infusion on days 1-5. Concurrent chemotherapy was weekly cisplatin (30–40 mg/m^2^), and cisplatin on weeks 1, 4, and 7 (80–100 mg/m^2^) of IMRT. Moreover, palliative treatment, such as chemotherapy, intracavitary brachytherapy and salvage surgery, were provided whenever possible for patients who suffered relapse or metastatic disease during follow-up.


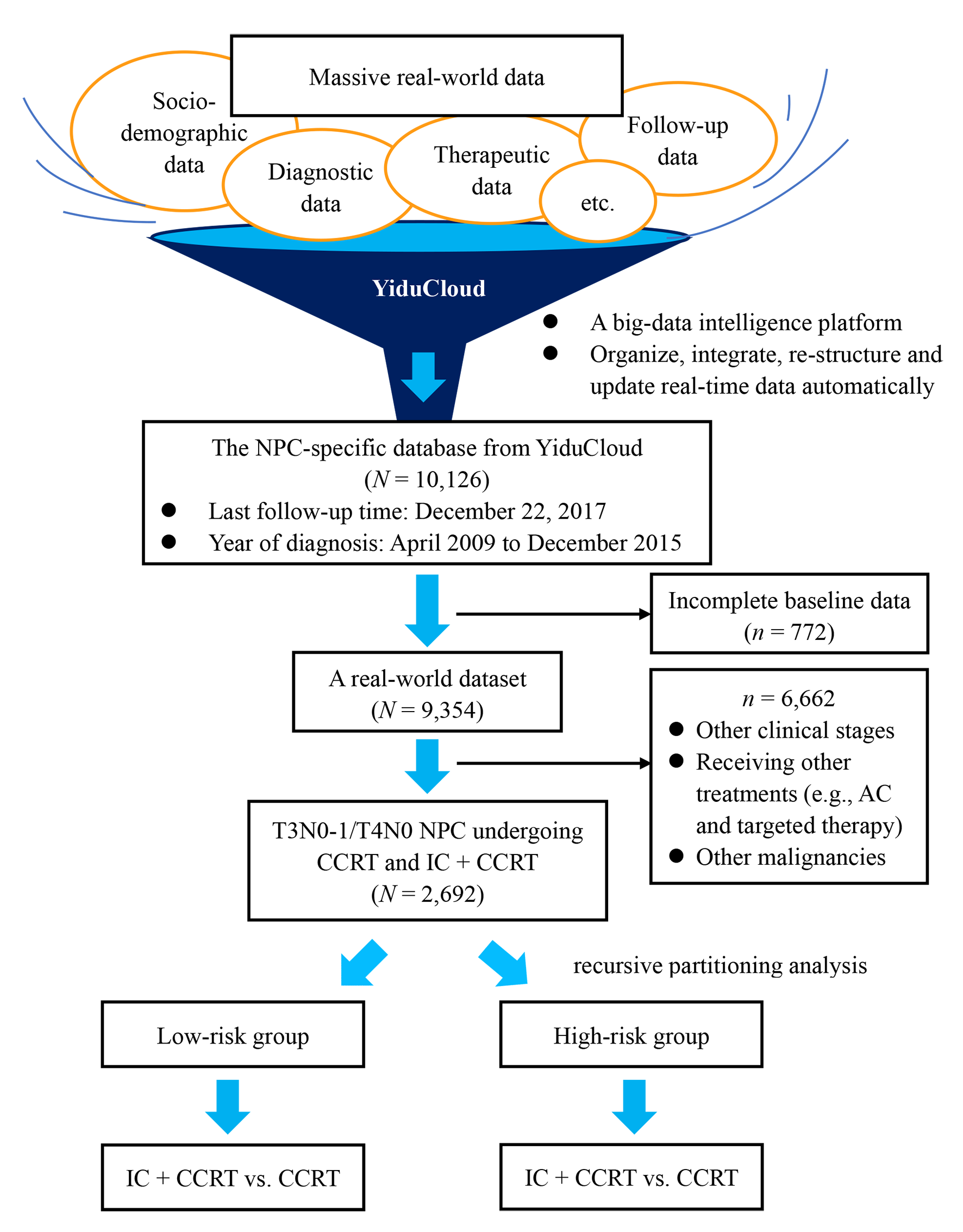


**Supplementary Figure S1.** The flow diagram depicting the study design. NPC = nasopharyngeal carcinoma; AC = adjuvant chemotherapy; CCRT = concurrent chemoradiotherapy; IC = induction chemotherapy.


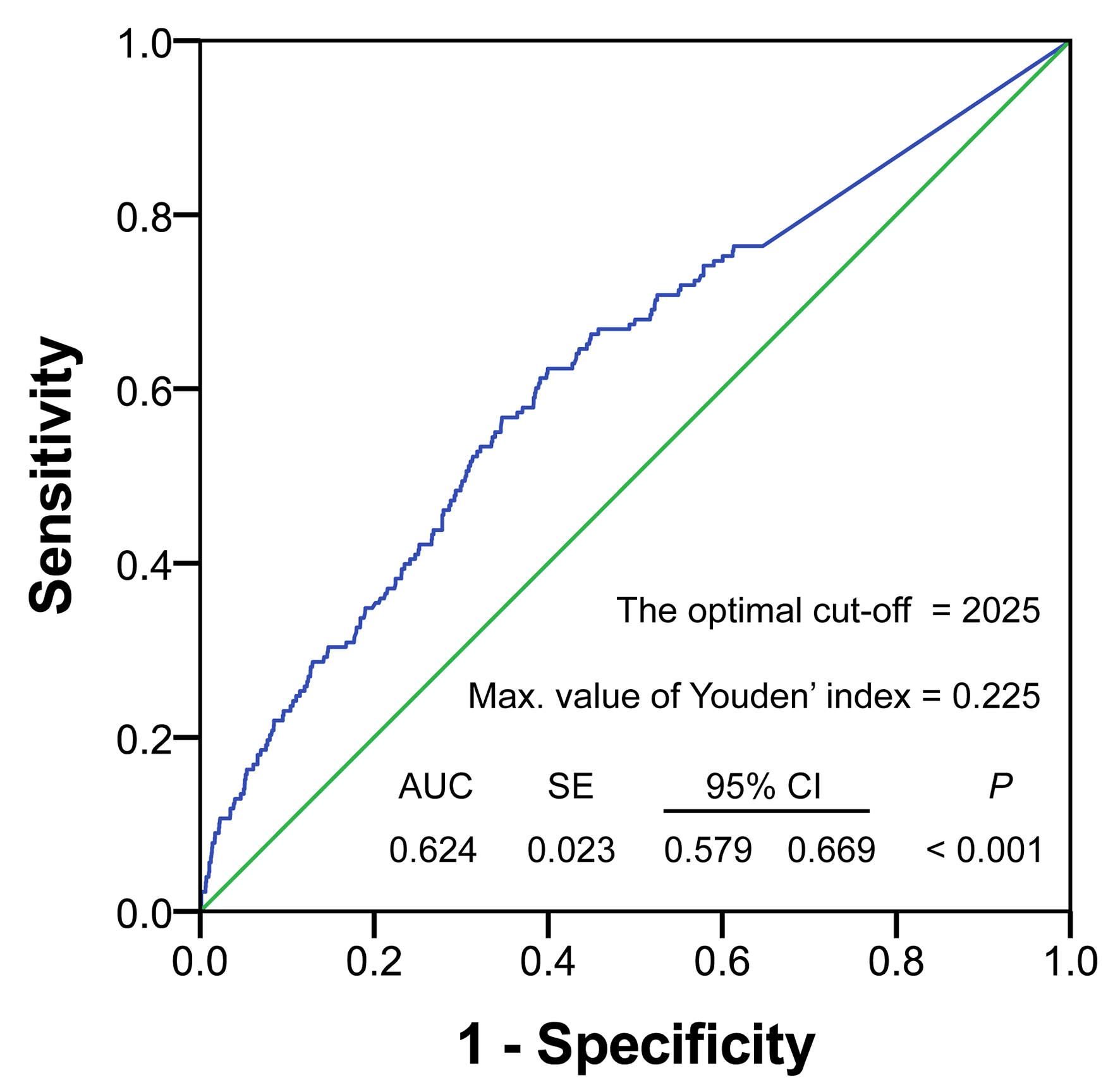


**Supplementary Figure S2.** Receiver-operating characteristic curve analysis of pre-treatment EBV DNA in patients with LANPC. The maximum value of Youden’s index indicates the optimal cut-off value. LANPC = locoregionally advanced nasopharyngeal carcinoma; EBV = Epstein-Barr virus; Max. = maximum; AUC = area under the curve; SE = sensitivity; CI = confidence interval.

**
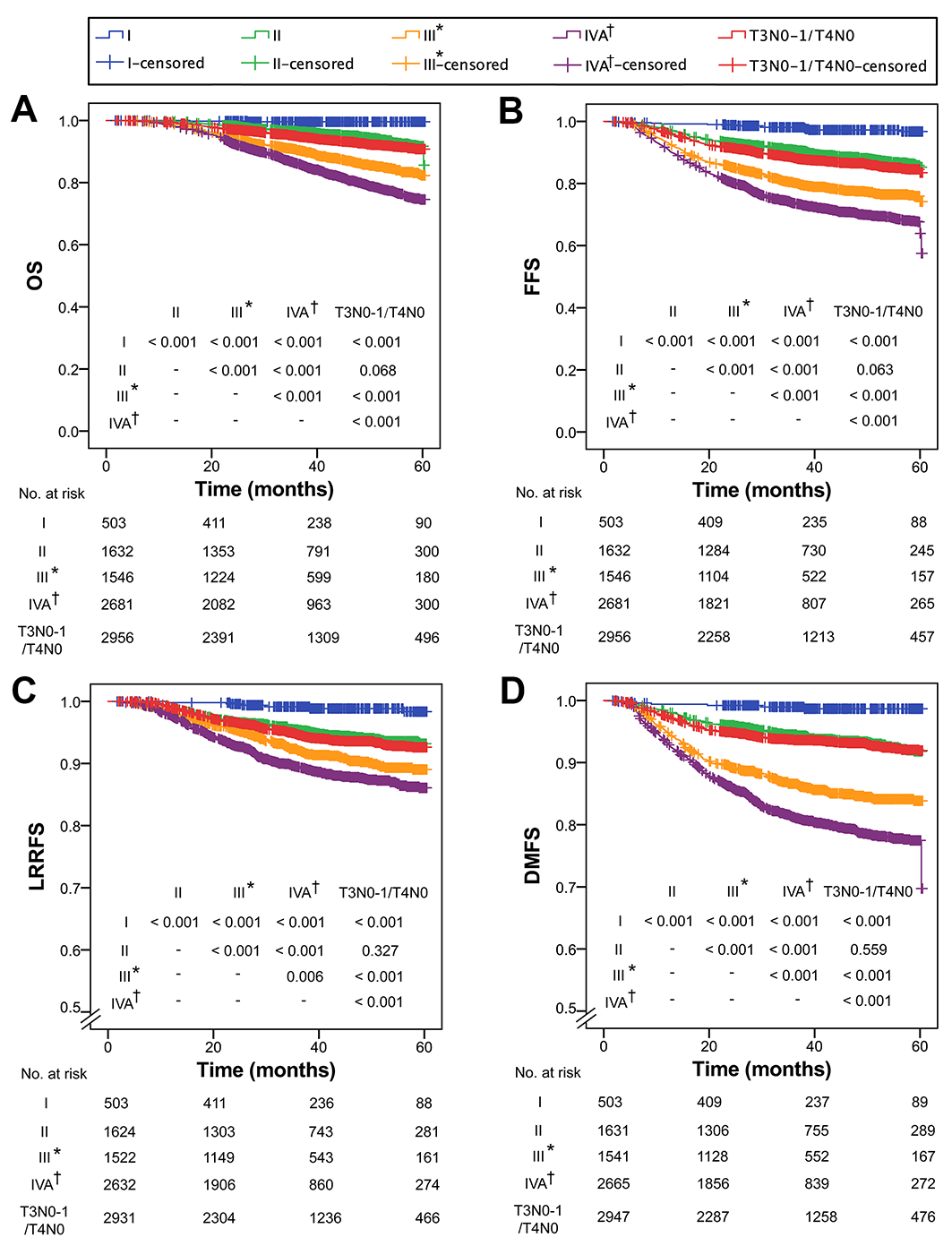
**

**Supplementary Figure S3.** Kaplan-Meier survival curves of patients with stage I, stage II, stage III without T3N0–1 (*), stage IVA without T4N0 (†), and T3N0–1/T4N0 NPC. NPC = nasopharyngeal carcinoma; OS = overall survival; FFS = failure-free survival; LRRFS = locoregional relapse-free survival; DMFS = distant metastasis-free survival.


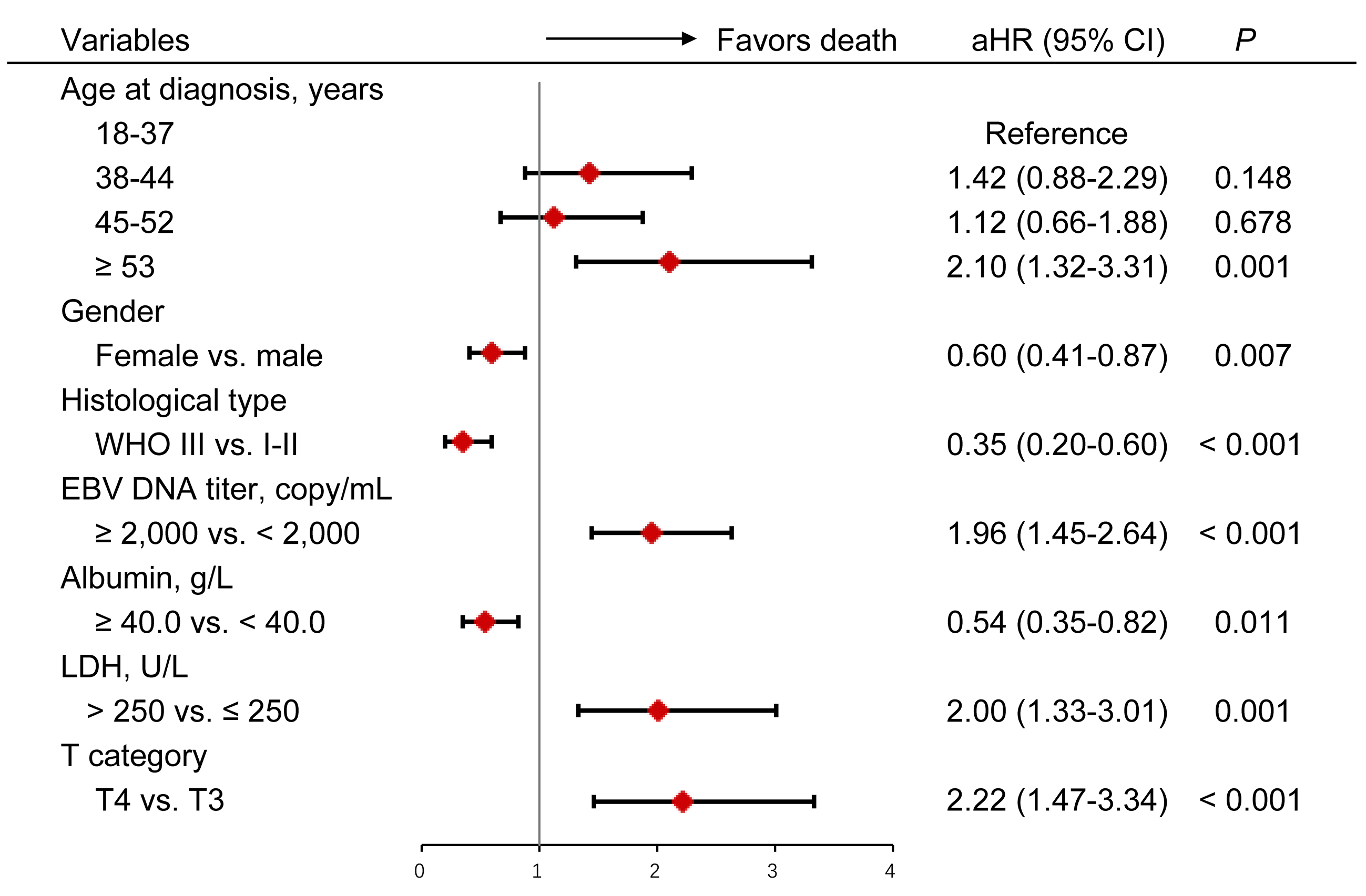


**Supplementary Figure S4.** Forest plots depicting adjusted hazard ratios (aHRs) and 95% confidence intervals (CIs) for multivariate analysis. Squares represent aHRs with 95% CIs indicated by horizontal bars. WHO = World Health Organization; EBV = Epstein-Barr virus; LDH = lactate dehydrogenase.


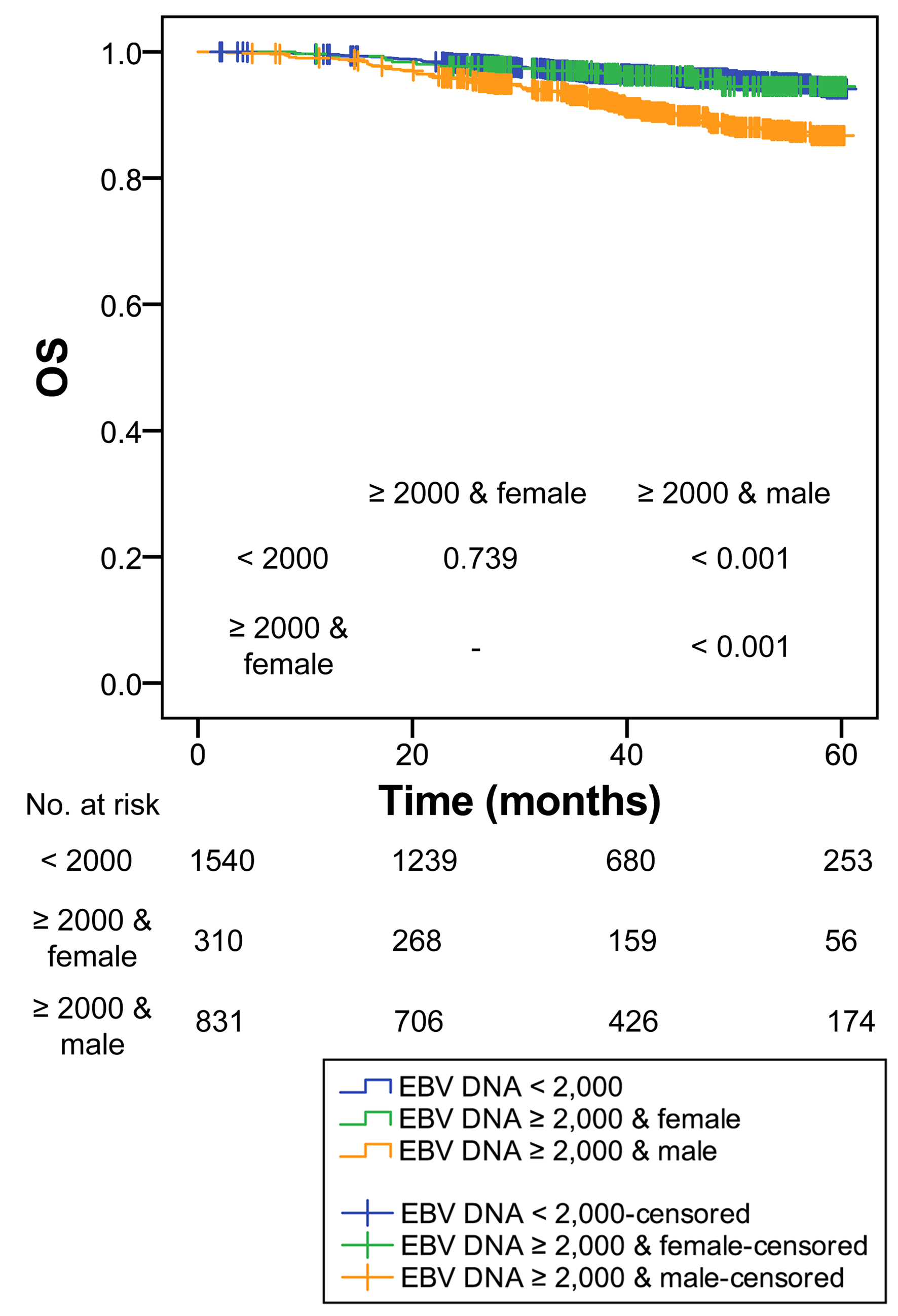


**Supplementary Figure S5.** Kaplan-Meier OS curves of three branches of RPA-generated risk stratification. RPA = recursive partitioning analysis; EBV = Epstein-Barr virus; OS = overall survival; No. = number.


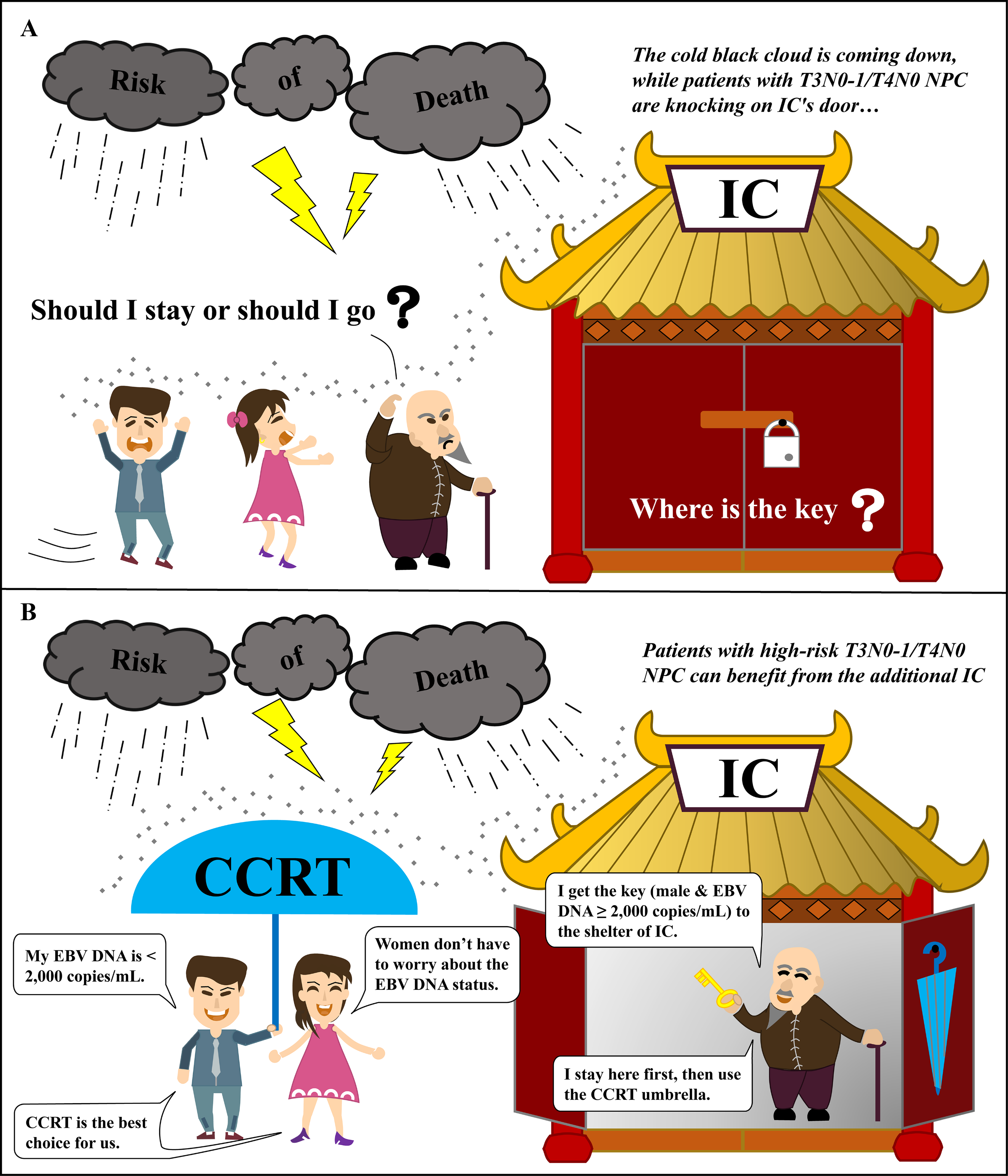


**Supplementary Figure S6.** The comics summarizing the question, results, and conclusions of the study. NPC = nasopharyngeal carcinoma; EBV = Epstein-Barr virus; CCRT = concurrent chemoradiotherapy; IC = induction chemotherapy.
